# Temporal trends in, and associations of, early-career general practitioner prescriptions of second-line Type 2 Diabetes medications, 2010–2018

**DOI:** 10.1371/journal.pone.0280668

**Published:** 2023-01-20

**Authors:** Irena Patsan, Amanda Tapley, Peter Davoren, Alison Fielding, Elizabeth Holliday, Jean Ball, Andrew Davey, Mieke van Driel, Rachel Turner, Katie Mulquiney, Neil Spike, Kristen FitzGerald, Parker Magin

**Affiliations:** 1 University of Newcastle, School of Medicine and Public Health, Callaghan, NSW, Australia; 2 GP Synergy, NSW & ACT Research and Evaluation Unit, Regional Training Organisation, Mayfield West, NSW, Australia; 3 Griffith University, Southport, QLD, Australia; 4 Diabetes and Endocrinology, Gold Coast Hospital, Southport, QLD, Australia; 5 Clinical Research Design and Statistical Support Unit (CReDITSS), Hunter Medical Research Institute (HMRI), New Lambton Heights, NSW, Australia; 6 Primary Care Clinical Unit, Faculty of Medicine, University of Queensland, Brisbane, Queensland, Australia; 7 Department of General Practice and Primary Health Care, University of Melbourne, Victoria, Australia; 8 Eastern Victoria General Practice Training (EVGPT), Regional Training Organisation, Hawthorn, Victoria, Australia; 9 School of Medicine, University of Tasmania, Hobart, Tasmania, Australia; 10 General Practice Training Tasmania (GPTT), Regional Training Organisation, Hobart, Tasmania, Australia; Shuguang Hospital, CHINA

## Abstract

**Introduction:**

Second-line pharmacotherapy for Type 2 Diabetes Mellitus (‘diabetes’) is necessary for optimal glycaemic control and preventing longer-term complications. We aimed to describe temporal trends in, and associations of, Australian general practitioner (GP) registrars’ prescription, and initiation, of ‘new’ second-line oral agents (dipeptidyl peptidase 4 inhibitors, sodium-glucose cotransporter 2 inhibitors, glucagon-like peptide 1 agonists) compared to sulphonylureas.

**Materials and methods:**

A longitudinal analysis (2010–2018) of data from the Registrar Clinical Encounters in Training project. Analysis included any diabetes problem/diagnosis that involved prescription of sulphonylureas or ‘new’ oral agents. Simple and multiple logistic regression models were fitted within the generalised estimating equations framework.

**Results:**

2333 registrars recorded 6064 diabetes problems/diagnoses (1.4%). 835 problems/diagnoses involved sulphonylurea or ‘new’ medication prescription. Of these, 61.0% [95% CI:57.4–64.4] involved ‘new’ medication prescription. 230 problems/diagnoses involved sulphonylurea or ‘new’ medication initiation, with 77% [95%CI:70.8–82.1] involving a ‘new’ medication. There was a significant 52% per year increase in prescribing (OR = 1.52[95% CI:1.38–1.68],p<0.001), and a 77% per (two-to-three-year) time-interval increase in initiation (OR = 1.77,[95% CI:1.30–2.43],p = <0.001) of ‘new’ medications compared to sulphonylureas. ‘New’ medications were prescribed less for non-English-speaking patients. There was some regional variation in prescribing.

**Conclusion:**

Registrar uptake of ‘new’ oral agents compared to sulphonylureas has increased rapidly.

## Introduction

Type 2 Diabetes Mellitus (hereafter ‘diabetes’) is largely managed within primary care, where it is one of the most commonly encountered conditions [1]. It is recognised as a national health priority in Australia and internationally [1, 2]. In 2017–18, approximately one million Australians (4.1%) had diabetes [3], with an estimated annual cost impact of over $10 billion [4]. Diabetes can result in micro- and macro-vascular complications with increased risk of morbidity including cardiovascular disease, stroke, kidney failure, blindness and limb amputations, and mortality [1]. General practitioners (GPs) have a vital role in the provision of care, contributing to individual health, quality of life outcomes, and ultimately, the burden of disease attributable to diabetes.

While lifestyle measures, including dietary intake, physical activity and weight management are recognised as initial primary care management [1], pharmacological means are often necessary to achieve optimal glycaemic control and potentially prevent long-term complications [5]. The Australian Pharmaceutical Benefits Scheme (PBS) subsidises the costs of medicines for all Australian citizens, though not all available medicines are subsidised. The number of Australians receiving PBS pharmacotherapy for diabetes treatment is increasing and, of the 3.9% of Australians who were receiving diabetes medications during 2016, half were on multiple medications [6].

A patient-centred approach focusing on individual patient needs, clinical judgement, and selection of appropriate pharmacological management approach [1] is vital in diabetes management. Glycaemic efficiency, overall safety (i.e. risk of hypoglycaemia, weight gain, and comorbidities), route of administration (oral vs subcutaneous), medication cost, and patient age and preference, guide GPs in determining pharmacotherapy, informed by evidence and up-to-date clinical practice guidelines [1]. In Australia, clinical guidelines including those available from the Royal Australian College of General Practitioners (RACGP) [1], Diabetes Australia [7], and eTG Complete Therapeutic Guidelines [8] provide practitioners with an individualised, stepwise, and progressive approach to pharmacological treatment. Internationally, comparable resources include the UK National Institute for Health and Care Excellence (NICE) guidelines [9] and the American Diabetes Association Practice Guidelines [10].

Although guidelines provide GPs with consistent advice on first-line pharmacotherapy (metformin in most patients), there are some inconsistencies in choice of second-line oral agents. These recommendations have also shifted over the past several years in response to accumulating evidence on second-line hypoglycaemic efficacy and safety. Thus, for practitioners there may be some ambiguity around selection of the most appropriate second-line medication type for individual patients. More than one second-line glucose-lowering pharmacotherapy may be prescribed, usually in combination with metformin [1].

Traditionally, sulphonylureas are prescribed as second-line treatment, in combination with metformin [1]. Long-term evidence supports the safety and effectiveness of ‘old’ agents including metformin, sulphonylureas, and insulin for health outcomes, including significant decreases in microvascular complications and, in some instances, mortality [11–13]. ‘Old’ hypoglycaemic medications may also have a ‘legacy’ effect with prevention of long-term vascular complications [11–13]. Sulphonylureas and insulin, however, have been shown to increase the risk of hypoglycaemia [11, 12].

In recent years, there has been an expansion of available diabetes hypoglycaemic medications. ‘New’ diabetes medications, including sodium-glucose cotransporter 2 inhibitors (SGLT-2-I), glucagon-like peptide 1 agonists (GLP-1-A), and dipeptidyl peptidase 4 inhibitors (DPP-4-I), improve glycaemic control [5]. These newer agents have become PBS-subsidised over the past several years, but with restrictive prescribing requirements [6]. Despite these restrictions, there has been uptake of the newer agents. In 2017, the percentages of total hypoglycaemic medications dispensed in Australia was: sulfonylurea 29%; DPP-4-I 28%; GLP-1-A 4.5%, SGLT-2-I 15% [14]. This uptake has also been seen in other, comparable, health systems internationally: in England the percentages were 35%, 24%, 5.6%, and 10%, respectively; in Scotland, 28%, 19%, 3.40%, and 10.10%; and in Canada, 29%, 21%, 1.9%, and 12% [14].

Increasing evidence suggests these medicines have lower risk of hypoglycaemia than sulphonylureas and insulin, promote weight loss or are weight neutral [1], and reduce cardiovascular outcomes [15] and mortality in patients at increased cardiovascular risk [16]. These outcomes have been established in the short-to-medium term but more evidence is required to understand long-term effects [15, 17].

While having generally favourable adverse-effect profiles, ‘new’ oral agents have potential adverse effects: diabetic ketoacidosis and genitourinary infections with SGLT-2-I use [18, 19]; gastrointestinal side effects and possibly pancreatitis with GLP-1-As [18]; and increased risk of hospitalization for heart failure with DPP-4-Is [18, 20]. Also, newer agents are considerably more expensive to the PBS, and the patient co-payment is higher, than for sulphonylureas.

The rapid expansion of medications available for diabetes management in recent years has added to complexity in pharmacological treatment algorithms. These algorithms are especially important in supporting GPs’ selection of appropriate treatment. Little is known about early-career GP registrars’ (doctors undertaking specialist vocational training in general practice) clinical practice regarding choice of second-line medications. Prescribing patterns are being established during the steep learning curve of GP vocational training. Thus, it is important to understand what factors are associated with their prescribing choices. Registrars comprise approximately 10% of the total Australian GP workforce by headcount [21, 22], and therefore contribute significantly to the management of patients with diabetes.

In this study, we aimed to describe temporal trends in GP registrar prescription of, and initiation of, ‘new’ second-line oral agents compared to sulphonylureas for the management of diabetes from 2010–2018, and to examine associations regarding this prescription choice.

## Materials and methods

### Setting

The study was conducted within Australian specialist general practice vocational training as part of the Registrar Clinical Encounters in Training (ReCEnT) project. Doctors seeking to specialize in general practice complete three-to-four years of full-time equivalent training (as ‘registrars’) through the Australian General Practice (AGPT) training program. For most registrars, at least 18 months full-time equivalent hours of training must be in a general practice setting. Education is delivered via Regional Training Organizations (RTOs) (or Regional Training Providers (RTPs) prior to 2016, when structural changes were made to the delivery of the AGPT program). GP registrars from five (of 17) Australian RTPs and three (of nine) Australian RTOs participated in ReCEnT during 2010–2015 and 2016–2018, respectively. The RTPs/RTOs are/were situated in five states and a territory.

ReCEnT is an ongoing, multicentre cohort study of GP registrars’ in-consultation clinical and educational practice. As previously detailed [23], registrars complete three rounds of data collection over three general practice-based training terms, in each term documenting 60 consecutive consultations. Information collected encompasses registrar, practice, patient, consultation, and educational factors. This includes problems/diagnoses managed and medicines prescribed. Data are intended to reflect a ‘usual week of general practice’.

Only consultations in the general practice office setting are recorded. Consultations in other settings and specialized clinics (nursing home visits, home visits, immunization clinics etc.) are excluded.

Data collection is a routine component of registrars’ educational programs, and they receive an individualized feedback report detailing their clinical practice with comparisons to their peers’ practice. Registrars may also provide consent for this data to be used for research purposes.

### Scope of the analysis

The analysis included data of any diabetes problem/diagnosis that entailed prescription of either a sulphonylurea or a ‘new’ medication. For this analysis, ‘diabetes’ included problems/diagnoses recorded specifically as Type 2 diabetes mellitus or non-insulin dependent diabetes mellitus, and also problems/diagnoses recorded as ‘diabetes mellitus’ without reference to type of diabetes. The assumption made was that these would be predominantly Type 2 diabetes. Problems/diagnoses in ReCEnT are coded to the International Primary Care Classification (ICPC-2) [24], and diabetes was defined utilising ICPC-2 codes detailed in Appendix 1 in S1 File. The study did not include codes for ‘pre-diabetes’. Only consultations with patients aged ≥16-years were included in the analysis.

### Outcome factor

The outcome factor was whether the medication prescribed was a sulphonylurea or a ‘new’ medication (i.e. DPP-4-I, SGLT-2-I, or GLP-1-A, or a combination of one of these with metformin). In ReCEnT, medications prescribed are coded according to the Anatomical Therapeutic Chemical (ATC) classification [25]. ATC codes included in our analysis are presented in Appendix 2 in S1 File. If a sulphonylurea and a ‘new’ medication were prescribed in the same consultation, that consultation was excluded from analysis.

In a secondary analysis, only newly-prescribed medications (that is, oral agents initiated at the index consultation) were included in the analysis.

### Study factor

The variable of interest for the analysis of temporal trends in prescribing was ‘year’ of consultation.

For the secondary analysis involving only initiated medications, year of consultation was aggregated (see below, ‘statistical analyses’).

### Independent variables

Other independent variables of interest in establishing associations of prescribing a ‘new’ medication rather than a sulphonylurea were patient, registrar, practice, and consultation factors (see Table 2 for the individual independent variables).

### Statistical analyses

Analysis was performed on 18 rounds of ReCEnT data collection from 2010–2018 and was conducted at the individual problem/diagnosis level (rather than consultation level).

Registrar problems/diagnoses involving diabetes, and proportions of these problems/diagnoses involving prescription of different medication classes, were calculated with 95% confidence intervals (CIs) using standard errors adjusted for repeated measures within registrars. Descriptive statistics included frequencies for categorical variables and mean with standard deviations for continuous variables. The frequencies of categorical variables were compared between outcome categories using Chi-squared tests, or Fisher’s exact test when the expected cell count was low. The means of continuous variables were compared using t-tests.

To test for temporal trends in the prescribing of ‘new’ medications compared to a sulphonylurea, and to test for associations of diabetes medications prescribed being a ‘new’ medication rather than a sulphonylurea, simple and multiple logistic regression models were fitted within the generalised estimating equations (GEE) framework to account for repeated measures within registrars. An exchangeable working correlation structure was assumed. Univariate analyses were conducted for each covariate and were repeated including year of consultation (Appendix 3 in S1 File). Covariates with a p-value <0.20 in either of the two univariate analyses were considered for inclusion within the multiple regression model. Fitted covariates with a p-value >0.20 in the multivariable model were tested for removal, and if this resulted in a substantively unchanged model (defined as changes in remaining odds ratios of <10%), the covariate was removed from the final model.

For the secondary analysis including only medications initiated at the index consultation, due to smaller numbers of prescribed medications, year of consultation was aggregated to create four levels (Level 1: 2010/2011/2012, Level 2: 2013/2014, Level 3: 2015/2016 and Level 4: 2017/2018).

In a post hoc analysis, we calculated proportion of registrar consultations being diabetes in the 45-64-year patient age group to enable comparison to previously published literature.

Statistical analyses were programmed using STATA 14.1 and SAS V9.4.

### Ethics

ReCEnT study approval is by the University of Newcastle’s Human Research Ethics Committee (Reference: H-2009-0323).

## Results

The analysis included 2333 participating GP registrars (response rate 96.0%), 5470 registrar-rounds of data collection, 266,644 consultations, and 423,769 problems/diagnoses. See Table 1 for demographics of participating registrars and their practices.

**Table 1:** Demographics of participating GP registrars and their practices.

Registrar variables (n = 2333)		n (%)
Registrar gender	Male	866 (37.1)
Qualified as a doctor overseas	Yes	447 (19.3)
Pathway registrar enrolled in	General	1647 (71.3)
	Rural	663 (28.7)
**Registrar round/practice variables (n = 5470)**		
Registrar age (years)	Mean ± SD	32.5 ± 6.3
Registrar works part-time	Yes	1725 (76.2)
Registrar training term	Term 1	1845 (79.1)
	Term 2	319 (13.7)
	Term 3	169 (7.2)
Practice rurality	Major city	1351 (58.5)
	Inner regional	619 (26.8)
	Outer regional remote	340 (14.7)
Practice SEIFA^†^ index	Mean ± SD	5.3 ± 2.8
Practice routinely bulk bills	Yes	664 (28.7)
Practice size	Small (1–5 GPs^‡^)	915 (50.5)
	Large (6–10+ GPs^‡^)	1343 (59.5)

^†^Socio-economic Indexes for Areas (SEIFA)

^‡^General practitioners (GPs)

Diabetes problems/diagnoses comprised 1.4% [95% CI:1.3–1.4] (n = 6064) of all problems/diagnoses and were managed in 1.8% [95% CI:1.8–1.9] of consultations. Of consultations in patients aged 45-64-years, 3.3% involved diabetes problems/diagnoses.

A total of 835 diabetes problems/diagnoses entailed prescription of sulphonylureas or ‘new’ second-line hypoglycaemic medications, without co-prescription (there were 81 additional problems/diagnoses where sulphonylureas and ‘new’ medications were prescribed concurrently). Of the 835 included problems/diagnoses, 39.0% [95% CI:35.6–42.6] (n = 326) involved prescription of a sulphonylurea, and 61.0% [95% CI:57.4–64.4] (n = 509) involved a prescription of a ‘new’ medication at the index consultation.

The oral agents prescribed and initiated are listed in Appendix 4 in S1 File. The most commonly prescribed/initiated sulphonylurea was ‘gliclazide’. For ‘new’ medications, ‘metformin and sitagliptin’, ‘exenatide’, ‘sitagliptin’, and ‘empagliflozin’ were most commonly prescribed/initiated.

Table 2 details univariate characteristics associated with registrar prescription of second-line diabetes medications, adjusted by year.

**Table 2:** Characteristics associated with prescribing medications for T2DM (adjusted by year).

			Diabetes medications		
Factor group	Variable	Class	Sulphonylureas	‘New’ agents	p
Patient factors	Patient age group	16–49	57 (18%)	101 (20%)	0.12
		50–64	125 (39%)	202 (40%)	
		65+	140 (43%)	196 (39%)	
	Patient gender	Male	162 (51%)	263 (54%)	0.83
		Female	153 (49%)	228 (46%)	
	Aboriginal Torres Strait Islander	No	307 (98%)	467 (98%)	0.35
		Yes	5 (2%)	11 (2%)	
	NESB^†^	No	268 (86%)	419 (87%)	0.068
		Yes	44 (14%)	64 (13%)	
	Patient/practice status	Existing patient	169 (52%)	281 (56%)	0.34
		New to registrar	143 (44%)	198 (40%)	
		New to practice	10 (3%)	22 (4%)	
Registrar factors	Registrar gender	Male	154 (47%)	222 (44%)	0.044
		Female	172 (53%)	287 (56%)	
	Registrar full-time or part-time	Part-time	60 (19%)	102 (21%)	0.28
		Full-time	257 (81%)	389 (79%)	
	Term	Term 1	127 (39%)	191 (38%)	0.44
		Term 2	112 (34%)	197 (39%)	
		Term 3	87 (27%)	121 (24%)	
	Worked at practice previously	No	243 (76%)	369 (73%)	0.21
		Yes	78 (24%)	136 (27%)	
	Qualified as doctor in Australia	No	65 (20%)	115 (23%)	0.15
		Yes	261 (80%)	393 (77%)	
	Registrar age	mean (SD)	33 (7)	33 (6)	0.79
Practice factors	Practice size^‡^	Small (<2–4)	139 (44%)	220 (45%)	0.16
		Large (5–10+)	178 (56%)	269 (55%)	
	Practice routinely bulk bills	No	252 (78%)	336 (66%)	0.26
		Yes	72 (22%)	170 (34%)	
	Rurality	Major city	175 (55%)	246 (49%)	0.007
		Inner regional	78 (24%)	163 (33%)	
		Outer regional remote	67 (21%)	90 (18%)	
	Region^§^	Region 1	74 (23%)	127 (25%)	<0.001
		Region 2	35 (11%)	27 (5%)	
		Region 3	39 (12%)	77 (15%)	
		Region 4	139 (43%)	127 (25%)	
		Region 5	12 (4%)	10 (2%)	
		Region 6	18 (6%)	100 (20%)	
		Region 7	9 (3%)	41 (8%)	
	SEIFA index^¶^	mean (SD)	5 (3)	5 (3)	0.65
Consultation factors	New problem seen	No	280 (95%)	447 (96%)	0.17
		Yes	15 (5%)	17 (4%)	
	Sought help any source	No	284 (87%)	388 (76%)	0.015
		Yes	42 (13%)	121 (24%)	

^†^Non-English-speaking background (NESB)

^‡^Defined as how many GPs (full time equivalents) work at this practice

^§^Region is Regional Training Provider (Regional Training Organisation, or regional unit of a Regional Training Organisation)

^¶^Socio-economic Indexes for Areas (SEIFA)

There were 230 diabetes problems recorded where a sulphonylurea or ‘new’ medication (without co-prescription) was initiated. Of these problems, 77.0% [95% CI:70.8–82.1] (n = 177) involved initiation of a ‘new’ medication.

### Temporal trends in prescribing and initiation of second-line diabetes medicines

**Fig 1:**
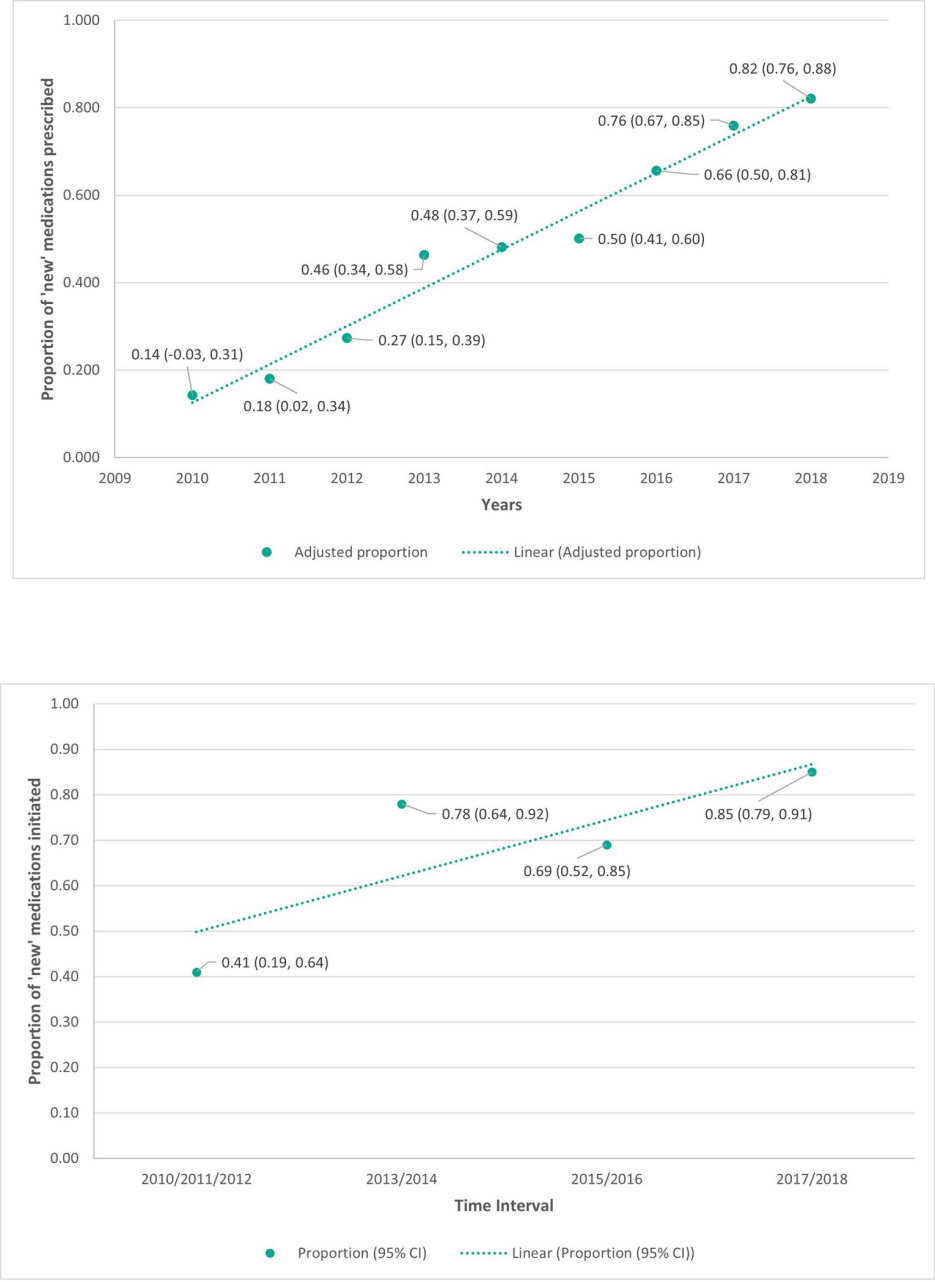
a. Adjusted proportion (with 95% CI) of ‘new’ diabetes medications compared to sulphonylureas prescribed from 2010–2018. b. Adjusted proportion (with 95% CI) of ‘new’ diabetes medications compared to sulphonylureas initiated over time from 2010–2018.

Fig 1A shows adjusted estimates of the temporal trend in proportion of ‘new’ medications prescribed compared to sulphonylureas. A timeline of important contextual influences on prescribing can be seen in Appendix 5 in S1 File. In the adjusted logistic regression model (Table 3) there was a significant 52% increase in prescribing of ‘new’ medications compared to sulphonylureas for each subsequent year (OR = 1.52 [95% CI:1.38–1.68], p<0.001).

**Table 3:** Multivariable associations of GP registrar prescription of ‘new’ diabetes medications compared to sulphonylureas over time (2010–2018).

			Univariate		Adjusted	
Factor group	Variable	Class	OR (95% CI)	p	OR (95% CI)	p
Intervention factors	Year of consult		1.49 (1.39, 1.61)	<0.001	1.52 (1.38, 1.68)	<0.001
Patient factors	Patient age group	50–64	0.90 (0.61, 1.34)	0.62	0.80 (0.47, 1.38)	0.43
	Referent: 16–49	65+	0.82 (0.55, 1.21)	0.31	0.62 (0.36, 1.05)	0.078
	Aboriginal Torres Strait Islander	Yes	1.47 (0.52, 4.14)	0.47	3.33 (0.76, 14.5)	0.11
	NESB^†^	Yes	0.92 (0.62, 1.38)	0.70	0.48 (0.28, 0.82)	0.008
Registrar factors	Registrar gender	Female	1.12 (0.83, 1.51)	0.45	1.33 (0.92, 1.93)	0.13
Practice factors	Practice size^‡^	Small (<2–4)	1.07 (0.80, 1.44)	0.63	0.69 (0.47, 1.02)	0.064
	Practice routinely bulk bills	Yes	1.82 (1.30, 2.57)	<0.001	1.55 (0.93, 2.59)	0.094
	Rurality (referent: Major city)	Inner regional	1.51 (1.07, 2.14)	0.021	1.50 (0.92, 2.44)	0.10
		Outer regional remote	0.99 (0.67, 1.47)	0.98	1.10 (0.55, 2.22)	0.79
	Region^§^	Region 2	0.46 (0.26, 0.79)	0.005	0.92 (0.39, 2.15)	0.85
	(referent: Region 1)	Region 3	1.21 (0.71, 2.07)	0.48	1.11 (0.55, 2.23)	0.78
		Region 4	0.55 (0.37, 0.81)	0.0023	0.58 (0.35, 0.96)	0.035
		Region 5	0.55 (0.20, 1.53)	0.25	0.58 (0.16, 2.18)	0.42
		Region 6	3.33 (1.91, 5.79)	<0.001	1.57 (0.66, 3.78)	0.31
		Region 7	2.71 (1.27, 5.78)	0.010	0.57 (0.22, 1.44)	0.23
Consultation factors	New problem seen	Yes	0.66 (0.33, 1.31)	0.23	0.54 (0.21, 1.36)	0.19
	Sought help any source	Yes	1.98 (1.36, 2.88)	<0.001	1.43 (0.90, 2.30)	0.13

^†^Non-English-speaking background (NESB)

^‡^Defined as how many GPs (full time equivalents) work at this practice

^§^Region is Regional Training Provider / Regional Training Organisation (or regional unit of a Regional Training Organisation)

Fig 1B shows adjusted estimates of the temporal trend in proportion of initiated ‘new’ medications compared to sulphonylureas. In the adjusted logistic regression model (Table 4), initiation of a ‘new’ medications compared to prescription of a sulphonylurea increased significantly with time (OR = 1.77, [95% CI:1.30–2.43], p = <0.001 with each increase in time interval (2010/11/12; 2013/14; 2015/16; and 2017/18).

**Table 4:** Multivariable associations of GP registrar initiation of ‘new’ diabetes medications compared to sulphonylureas over time (2010–2018).

			Univariate		Adjusted	
Factor group	Variable	Class	OR (95% CI)	p	OR (95% CI)	p
Intervention factors	Time period of consult		1.73 (1.29, 2.32)	<0.001	1.77 (1.30, 2.43)	<0.001
Registrar factors	Registrar full-time or part-time	Full-time	0.99 (0.47, 2.09)	0.98	0.56 (0.24, 1.32)	0.18
Consultation factors	New problem seen	Yes	0.33 (0.13, 0.82)	0.018	0.25 (0.10, 0.65)	<0.005

### Associations of prescribing and initiation of second-line diabetes medicines

Multivariable associations of GP registrar prescription and initiation of ‘new’ medications compared to sulphonylureas over time are presented in Tables 3 and 4, respectively.

The prescription of ‘new’ medications was less likely when the patient was of non-English-speaking background (OR = 0.48 [95% CI: 0.28, 0.82], p = 0.008). Limited variability in prescription patten was observed within practice training regions. However, one region did have an OR of 0.58 ([95% CI: 0.35, 0.96] p = 0.035) for prescribing ‘new’ medications when compared to the referent region. There was some evidence for Aboriginal and/or Torres Strait Islander patients being more likely to be prescribed a ‘new’ medication (OR 3.33 [95% CI: 0.76, 14.5], p = 0.11).

## Discussion

### Summary of main findings and relation to previous research

Prevalence of diabetes in registrars’ training: The prevalence of registrar experience with diabetes appears to be modest at 1.4% of all problems/diagnoses seen. Registrars in our study appear to see diabetes less than established GPs, with 3.3% of patients being seen for diabetes in the 45-64-year group, compared to 5.1% seen by GPs in a comparable study [26]. This is consistent with our previous findings suggesting that Australian registrars encounter patients with chronic disease less than established GPs [27].

Longitudinal findings: We found a significant 52% annual increase in the prescription and 77% increase in the initiation of ‘new’ oral agents per two-or-three-year time interval compared to sulphonylureas from 2010–2018. By 2018, the adjusted proportion of ‘new’ oral agents prescribed was 0.82 (Fig 1A), and of ‘new’ oral agents initiated was 0.85 (Fig 1B). While the findings are not directly comparable, it appears that the uptake of ‘new’ oral agents (when compared to prescription of sulfonylureas) may be greater by Australian GP registrars than by Australian established GPs (and by established GPs in some other countries) [14].

Associations of prescribing and initiating second-line oral agents: We identified several associations of GP registrar prescription and initiation of ‘new’ second-line oral agents compared to sulphonylureas. Most notably, the association of ‘new’ medication prescriptions being 48% less likely in patients of non-English-speaking background. There was also evidence for differences of ‘new’ medication versus sulphonylurea prescriptions in different regions, and some evidence for Aboriginal and/or Torres Strait Islander patients (p = 0.11) being more likely to be prescribed a ‘new’ medication.

### Interpretation and implications for education and further research

The significant increase in ‘new’ medication prescription compared to sulphonylureas may have important implications for GP vocational training. Appropriate prescription for individual patient needs within individually applied treatment algorithms is an important skill to be acquired in general practice training. Our findings reflect the increased options of second-line treatments beyond traditional sulfonylureas (including PBS subsidisation, as well as availability, in Australia) and the accumulating evidence of improved clinical cardiovascular outcomes with treatment with ‘new’ medications [18], at least in those with pre-existing increased cardiovascular risk [28]. However, consideration of the temporality of prescribing and the timelines of contextual factors (Appendix 5 in S1 File) suggests registrars’ ‘new’ hypoglycaemic prescribing may frequently have been anticipatory of, rather than in response to, guideline recommendation or Level I evidence. For example, in 2010–2012, when limited evidence existed for cardiovascular outcomes for ‘new’ medications, with very limited guideline recommendations for their use, we found 41% of second-line oral agent initiations were for ‘new’ medications (Fig 1B).

This is a striking exception to the usually slow uptake of evidence into clinical practice [29]. It is relevant that diabetes is highly prevalent, is managed primarily in general practice, and is associated with considerable morbidity and mortality [1]. Thus, GPs may be expected to be highly receptive to evidence, and amenable to change, in this area–potentially ahead of medium- and long-term evidence of mortality and major morbidity outcomes. It should also be noted that ‘new’ oral agents have been heavily promoted commercially, particularly for their role in weight loss [5], and this may also be a factor in their uptake [30].

The implication for GP vocational training (and GP continuing medical education) is that, despite documented generally slow uptake of evidence into practice [29], prompt and substantive change in practice is very much practicable. But this change, if ahead of convincing long-term evidence, may not necessarily benefit patients. With diabetes, an example is the potential for patient harms which emerged with use of thiazolideinediones [31]. Further research into how GPs, especially early-career GPs, integrate information sources into management practices is required to inform educational practice.

The finding that ‘new’ medications were less likely to be prescribed to patients of non-English-speaking backgrounds may be consistent with evidence that suggests language barriers may hinder doctor-patient communication with patients with diabetes [32]. It is possible this could create a barrier in discussing with patients the rationale for changing, and implementing that change, from existing long-term therapy with a sulphonylurea to a ‘new’ hypoglycaemic.

### Strengths and limitations

Our longitudinal methods allowed insight into temporal changes of registrar prescription of ‘new’ second-line oral agents versus sulphonylureas during vocational training. The ReCEnT project is currently the largest study of general practice trainees worldwide. This study covers a large geographical area across Australia including city, regional, remote, and very remote communities. Therefore, generalisability is strong for Australian GP vocational training, and findings may be relevant to international GP vocational training with similar training frameworks. However, while non-Australian training frameworks might often be similar (apprenticeship-style models of training), the healthcare system context across the different countries in which the training program is nested may have relevant differences (for example, medication availability and cost to patient).

The principal limitations of this study were lack of access to data on full patient medicines regimens, prescribing history, co-morbidities/cardiovascular risk, and patient convenience and preference for type of ‘new’ second-line medication prescribed. Another limitation was that medication availability was not practicable to include as a covariate in the multivariable model, however Appendix 5 in S1 File provides a timeline detailing this in the context of the Australian healthcare system. Also, despite the large number of covariates included in our multivariable analyses, there may be further unmeasured confounding (for example, changes in ‘new’ oral agent costing).

A further limitation is that some included ICPC-2 diabetes codes were not Type 2-specific. We are confident, however, that our classification of these as diabetes was accurate in the great majority of cases.

## Conclusions

Prevalence of Type 2 diabetes and range of hypoglycaemic treatment options have significantly increased over time. While metformin remains the optimal first-line option, uptake and choice of newer second-line oral agents compared to sulphonylureas is rapidly increasing.

This has potential to result in improved patient outcomes but increasing uptake may have been in advance of dissemination of evidence for improved outcomes and incorporation into evidence-based guidelines. This suggests a need for critical evaluation of the processes whereby rapid uptake of the ‘new’ medications has occurred. The implications of this for future clinical and educational practice should be further assessed.

## Supporting information

S1 File (DOCX)
