# Supplementary material for: Temporal trends in, and associations of, early-career general practitioner prescriptions of second-line Type 2 Diabetes medications, 2010–2018
Source: PLoS One. 2023 Jan 20;18(1):e0280668. doi: 10.1371/journal.pone.0280668 (PMC9858089; doi:10.1371/journal.pone.0280668)
Supplement: S1 File — (DOCX) [file pone.0280668.s001.docx]

**Supporting information**

**Appendix 1: Included International Primary Care Classification (ICPC-2) diagnostic codes**

| **Term_ab** | **ICPC_code** | **Term_code** | **Grouping**^†^ |
| --- | --- | --- | --- |
| Problem;foot/feet;diabetes | A29 | 30 | NS |
| Retinopathy;diabetic | F83 | 2 | NS |
| Neuropathy;diabetic | N94 | 12 | NS |
| Ulcer;diabetic | S97 | 13 | NS |
| Necrobiosis lipoidica diabetic | S99 | 31 | NS |
| Scleroedema;diabetic | S99 | 58 | NS |
| Check up;diabetes | T31 | 5 | NS |
| Test;diabetes review | T34 | 41 | NS |
| Advice/education;diabetes | T45 | 4 | NS |
| Consult;diabetes educator | T46 | 3 | NS |
| Admin;care plan;diabetes | T62 | 2 | NS |
| Referral;diabetes clinic | T67 | 3 | NS |
| Referral;diabetes education | T68 | 3 | NS |
| Reaction after;hypoglycaemia | T87 | 4 | NS |
| Hypoglycaemia | T87 | 5 | NS |
| Coma;hypoglycaemic | T87 | 1 | NS |
| Coma;insulin | T87 | 2 | NS |
| Diabetes;insulin dependent | T89 | 1 | NS |
| Diabetes;complicated | T89 | 4 | NS |
| Coma;diabetic | T89 | 5 | NS |
| Hyperglycaemia (diabetic) | T89 | 6 | NS |
| Ulcer;diabetic | T89 | 7 | NS |
| Diabetes;insulin dependent | T90 | 4 | NS |
| Diabetes;non insulin depend | T90 | 5 | T2DM |
| Diabetes;adult onset | T90 | 7 | T2DM |
| Diabetes;Type 2 | T90 | 9 | T2DM |
| Coma;diabetic | T90 | 1 | NS |
| Diabetes mellitus | T90 | 2 | NS |
| Diabetes;complicated | T90 | 3 | NS |
| Diabetes;Type 2;insulin treate | T90 | 16 | T2DM |
| Diabetes;complicated | T90 | 18 | NS |
| Nephropathy;diabetic | U88 | 11 | NS |

^†^Grouping Terms: NS – Diabetes Mellitus type not specified; T2DM – Type 2 Diabetes Mellitus

**Appendix 2: Included Anatomical Therapeutic Chemical (ATC) classification codes**

| **ATC code** | **ATC level name** | **Medication category**^†^ |
| --- | --- | --- |
| A10BB01  A10BB07  A10BB09  A10BB12  A10BD02  A10BD07  A10BD08  A10BD10  A10BD11  A10BD13  A10BD15  A10BD20  A10BH01  A10BH02  A10BH03  A10BH04  A10BH05  A10BJ01  A10BJ02  A10BJ05  A10BK01  A10BK02  A10BK03 | glibenclamide  glipizide  gliclazide  glimepiride  metformin and sulfonamides  metformin and sitagliptin  metformin and vildagliptin  metformin and saxagliptin  metformin and linagliptin  metformin and alogliptin  metformin and dapagliflozin  metformin and empagliflozin  sitagliptin  vildagliptin  saxagliptin  alogliptin  linagliptin  exenatide  liraglutide  dulaglutide  dapagliflozin  canagliflozin  empagliflozin | SU  SU  SU  SU  SU  DPP-4  DPP-4  DPP-4  DPP-4  DPP-4  SGLT-2  SGLT-2  DPP-4  DPP-4  DPP-4  DPP-4  DPP-4  GLP-1  GLP-1  GLP-1  SGLT-2  SGLT-2  SGLT-2 |

^†^Sulphonylureas (SU), dipeptidyl peptidase 4 inhibitors (DPP-4), sodium-glucose cotransporter 2 inhibitors (SGLT-2), glucagon-like peptide 1 agonists (GLP-1).

**Appendix 3: Characteristics associated with prescribing medications for diabetes (unadjusted)**

|  | | | **Diabetes medications** | | |
| --- | --- | --- | --- | --- | --- |
| **Factor group** | **Variable** | **Class** | **Sulphonylureas** | **‘New’ agents** | **p** |
| Patient factors | Patient age group | 16-49 | 57 (18%) | 101 (20%) | 0.58 |
|  |  | 50-64 | 125 (39%) | 202 (40%) |  |
|  |  | 65+ | 140 (43%) | 196 (39%) |  |
|  | Patient gender | Male | 162 (51%) | 263 (54%) | 0.53 |
|  |  | Female | 153 (49%) | 228 (46%) |  |
|  | Aboriginal Torres Strait Islander | No | 307 (98%) | 467 (98%) | 0.47 |
|  |  | Yes | 5 (2%) | 11 (2%) |  |
|  | NESB^†^ | No | 268 (86%) | 419 (87%) | 0.70 |
|  |  | Yes | 44 (14%) | 64 (13%) |  |
|  | Patient/practice status | Existing patient | 169 (52%) | 281 (56%) | 0.34 |
|  |  | New to registrar | 143 (44%) | 198 (40%) |  |
|  |  | New to practice | 10 (3%) | 22 (4%) |  |
| Registrar factors | Registrar gender | Male | 154 (47%) | 222 (44%) | 0.45 |
|  |  | Female | 172 (53%) | 287 (56%) |  |
|  | Registrar full-time or part-time | Part-time | 60 (19%) | 102 (21%) | 0.56 |
|  |  | Full-time | 257 (81%) | 389 (79%) |  |
|  | Term | Term 1 | 127 (39%) | 191 (38%) | 0.62 |
|  |  | Term 2 | 112 (34%) | 197 (39%) |  |
|  |  | Term 3 | 87 (27%) | 121 (24%) |  |
|  | Worked at practice previously | No | 243 (76%) | 369 (73%) | 0.42 |
|  |  | Yes | 78 (24%) | 136 (27%) |  |
|  | Qualified as doctor in Australia | No | 65 (20%) | 115 (23%) | 0.31 |
|  |  | Yes | 261 (80%) | 393 (77%) |  |
|  | Registrar age | mean (SD) | 33 (7) | 33 (6) | 0.71 |
| Practice factors | Practice size^‡^ | Small (<2-4) | 139 (44%) | 220 (45%) | 0.63 |
|  |  | Large (5-10+) | 178 (56%) | 269 (55%) |  |
|  | Practice routinely bulk bills | No | 252 (78%) | 336 (66%) | <0.001 |
|  |  | Yes | 72 (22%) | 170 (34%) |  |
|  | Rurality | Major city | 175 (55%) | 246 (49%) | 0.050 |
|  |  | Inner regional | 78 (24%) | 163 (33%) |  |
|  |  | Outer regional remote | 67 (21%) | 90 (18%) |  |
|  | Region^§^ | Region 1 | 74 (23%) | 127 (25%) | <0.001 |
|  |  | Region 2 | 35 (11%) | 27 (5%) |  |
|  |  | Region 3 | 39 (12%) | 77 (15%) |  |
|  |  | Region 4 | 139 (43%) | 127 (25%) |  |
|  |  | Region 5 | 12 (4%) | 10 (2%) |  |
|  |  | Region 6 | 18 (6%) | 100 (20%) |  |
|  |  | Region 7 | 9 (3%) | 41 (8%) |  |
|  | SEIFA index^¶^ | mean (SD) | 5 (3) | 5 (3) | 0.54 |
| Consultation factors | New problem seen | No | 280 (95%) | 447 (96%) | 0.23 |
|  |  | Yes | 15 (5%) | 17 (4%) |  |
|  | Sought help any source | No | 284 (87%) | 388 (76%) | <0.001 |
|  |  | Yes | 42 (13%) | 121 (24%) |  |

^†^Non-English-speaking background (NESB)

^‡^Defined as how many GPs (full time equivalents) work at this practice

^§^Region is Regional Training Provider (Regional Training Organisation, or regional unit of a Regional Training Organisation)

^¶^Socio-economic Indexes for Areas (SEIFA)

**Appendix 4: List of prescribed and initiated sulphonylureas and 'new' medications**

| **Prescribed sulphonylureas and ‘new’ medications** | **n (%)*** | **Initiated sulphonylureas and ‘new’ medications** | **n (%)** |
| --- | --- | --- | --- |
| gliclazide | 285 (33.3%) | gliclazide | 47 (20.4%) |
| metformin and sitagliptin | 149 (17.4%) | metformin and sitagliptin | 36 (15.7%) |
| exenatide | 72 (8.4%) | exenatide | 33 (14.3%) |
| sitagliptin | 72 (8.4%) | sitagliptin | 29 (12.6%) |
| empagliflozin | 52 (6.1%) | empagliflozin | 28 (12.2%) |
| dapagliflozin | 41 (4.8%) | dapagliflozin | 11 (4.8%) |
| linagliptin | 34 (4.0%) | metformin and dapagliflozin | 10 (4.3%) |
| glimepiride | 23 (2.7%) | metformin and empagliflozin | 7 (3.0%) |
| metformin and empagliflozin | 21 (2.5%) | linagliptin | 6 (2.6%) |
| metformin and vildagliptin | 18 (2.1%) | saxagliptin | 5 (2.2%) |
| metformin and dapagliflozin | 16 (1.9%) | glimepiride | 3 (1.3%) |
| saxagliptin | 16 (1.9%) | alogliptin | 3 (1.3%) |
| metformin and linagliptin | 10 (1.2%) | metformin and vildagliptin | 3 (1.3%) |
| glibenclamide | 7 (0.8%) | dulaglutide | 2 (0.9%) |
| metformin and sulfonamides | 7 (0.8%) | vildagliptin | 2 (0.9%) |
| metformin and saxagliptin | 7 (0.8%) | glibenclamide | 1 (0.4%) |
| vildagliptin | 7 (0.8%) | glipizide | 1 (0.4%) |
| metformin and alogliptin | 5 (0.6%) | metformin and sulfonamides | 1 (0.4%) |
| glipizide | 4 (0.5%) | canagliflozin | 1 (0.4%) |
| alogliptin | 4 (0.5%) | metformin and linagliptin | 1 (0.4%) |
| dulaglutide | 3 (0.4%) |  |  |
| canagliflozin | 1 (0.1%) |  |  |
| liraglutide | 1 (0.1%) |  |  |
| **Totals** | **855** |  | **230** |

*Note: Totals do not match number of problems as more than one ‘new’ medication could have been prescribed for one problem. If a sulphonylurea and a ‘new’ medication were prescribed in the same consultation, that consultation was excluded from analysis.*

**Appendix 5:** **‘New’ medications timeline including Australian Pharmaceutical Benefits Scheme (PBS), Clinical Practice Guidelines and Early evidence of cardiovascular/mortality outcomes.**

| **Year** | **Added to Australian Pharmaceutical Benefits Scheme** | **Added to Australian Clinical Practice Guidelines^†^** | **Early meta-analyses (Level I evidence) with evidence of improvement in any cardiovascular/mortality outcomes^β^** |
| --- | --- | --- | --- |
| **2008** | DPP-4^*^ (sitagliptin – in combination with metformin/sulphonylurea when HbA1c >7%) |  |  |
| **2010** | GLP-1^*^ (exenatide - dual and triple therapy)  DPP-4 (saxagliptin and vildagliptin – in combination with metformin/sulphonylurea when HbA1c >7%) | GLP-1 third line therapy (eTG) |  |
| **2011** |  | GLP-1 and DPP-4 third line therapy (RACGP) | Rater 2011 (GLP-1)^^^  Monami 2011 (GLP-1)^^^ |
| **2012** |  |  |  |
| **2013** | SGLT-2^*^ (dapagliflozin -dual therapy)  DPP4 (Alogliptin) |  |  |
| **2014** | DPP-4 (linagliptin, saxagliptin, sitagliptin, and vildagliptin – dual therapy) | GLP-1 and DPP-4 second line (eTG)  GLP-1, DPP-4, and SGLT-2 second and third line (RACGP) |  |
| **2015** | SGLT-2 (empagliflozin - dual therapy) |  |  |
| **2016** |  | SGLT-2 and DPP-4 alternative first line if metformin not tolerated, second and third line therapy (RACGP) | Tang 2016 (SGLT-2) **^‡^**^§Фα^  Wu JH 2016 (SGLT-2) **^‡^**^§Ф^  Savarese 2016 (SGLT-2 and DPP-4) **^‡^**^§Фψ^  Eriksson 2016 (DPP-4) **^‡^**^ψ^  Ding 2016 (GLP-1)**^‡^** |
| **2017** |  |  | Gargiulo 2017 (GLP-1)**^‡^**^ψ^ |
| **2018** |  |  |  |
| **2019** |  | SGLT-2 and DPP-4 second line, GLP-1 third line therapy (eTG) |  |

^*^Sodium-glucose cotransporter 2 inhibitors (SGLT-2); glucagon-like peptide 1 agonists (GLP-1); dipeptidyl peptidase 4 inhibitors (DPP-4).

^†^Therapeutic Guidelines (eTG) and Royal Australian College of General Practitioners (RACGP).

^^^No increase cardiovascular risk found; longer-term evidence needed

**^‡^**Improvement in Mortality

^§^Improvement in Myocardial infarction outcome

^#^Improvement in stroke outcome

^Ф^Improvement in heart failure outcome

^ψ^Improvement in composite Cardiovascular outcome

^α^Improvement in hospital admission outcome

**^β^**Appendix 5 references:

1. Ratner R, Han J, Nicewarner D, Yushmanova I, Hoogwerf BJ, Shen L. Cardiovascular safety of exenatide BID: an integrated analysis from controlled clinical trials in participants with type 2 diabetes. Cardiovasc. Diabetol. 2011; 10:22. doi:10.1186/1475-2840-10-22, 10.1186/1475-2840-10-22
2. Monami M, Cremasco F, Lamanna C, et al. Glucagon-like peptide-1 receptor agonists and cardiovascular events: a meta-analysis of randomized clinical trials. Exp Diabetes Res. 2011; 2011:215764. doi:10.1155/2011/215764, 10.1155/2011/215764
3. Tang H, Fang Z, Wang T, Cui W, Zhai S, Song Y. Meta-Analysis of Effects of Sodium-Glucose Cotransporter 2 Inhibitors on Cardiovascular Outcomes and All-Cause Mortality Among Patients With Type 2 Diabetes Mellitus. Am J Cardiol. 2016; 118(11):1774-1780. doi:10.1016/j.amjcard.2016.08.061
4. Wu, J. H. Y., et al. Effects of sodium-glucose cotransporter-2 inhibitors on cardiovascular events, death, and major safety outcomes in adults with type 2 diabetes: a systematic review and meta-analysis. Lancet Diabetes Endo 2016; 4(5): 411-419.
5. Savarese G., et al. Effects of Dipeptidyl Peptidase 4 Inhibitors and Sodium-Glucose Linked coTransporter-2 Inhibitors on cardiovascular events in patients with type 2 diabetes mellitus: A meta-analysis. Int J Cardiol 2016; 20:595-601. ISSN 0167-5273, <https://doi.org/10.1016/j.ijcard.2016.06.208>.
6. Eriksson JW, Bodegard J, Nathanson D, Thuresson M, Nyström T, Norhammar A. Sulphonylurea compared to DPP-4 inhibitors in combination with metformin carries increased risk of severe hypoglycemia, cardiovascular events, and all-cause mortality. Diabetes Res Clin Pr 2016; 117; 39-47. ISSN 0168-8227, <https://doi.org/10.1016/j.diabres.2016.04.055>.
7. Ding S., et al. Effect of glucagon-like peptide-1 on major cardiovascular outcomes in patients with type 2 diabetes mellitus: A meta-analysis of randomized controlled trials. Int J Cardiol 2016; 222; 957-962. ISSN 0167-5273, <https://doi.org/10.1016/j.ijcard.2016.07.199>.
8. Gargiulo, P., et al. Efficacy and safety of glucagon-like peptide-1 agonists on macrovascular and microvascular events in type 2 diabetes mellitus: A meta-analysis. Nutr Metab Cardiovas 2016. 27(12): 1081-1088.
